# Solving real-world optimization tasks using physics-informed neural computing

**DOI:** 10.1038/s41598-023-49977-3

**Published:** 2024-01-08

**Authors:** Jaemin Seo

**Affiliations:** Department of Physics, Chung-Ang University, Seoul, South Korea

**Keywords:** Applied mathematics, Computational science, Information technology, Information theory and computation

## Abstract

Optimization tasks are essential in modern engineering fields such as chip design, spacecraft trajectory determination, and reactor scenario development. Recently, machine learning applications, including deep reinforcement learning (RL) and genetic algorithms (GA), have emerged in these real-world optimization tasks. We introduce a new machine learning-based optimization scheme that incorporates physics with the operational objectives. This physics-informed neural network (PINN) could find the optimal path in well-defined systems with less exploration and also was capable of obtaining narrow and unstable solutions that have been challenging with bottom-up approaches like RL or GA. Through an objective function that integrates governing laws, constraints, and goals, PINN enables top-down searches for optimal solutions. In this study, we showcase the PINN applications to various optimization tasks, ranging from inverting a pendulum, determining the shortest-time path, to finding the swingby trajectory. Through this, we discuss how PINN can be applied in the tasks with different characteristics.

## Introduction

Optimization tasks are at the heart of many real-world applications across a variety of scientific disciplines, particularly in physics and engineering. From the seemingly simple task of swinging up a pendulum to the complex maneuver of a spacecraft swingby, these tasks demand a sophisticated and accurate understanding of various interconnected factors. At the core of an optimization task are three key components: governing laws, constraints, and goals. Governing laws, derived from physical principles, dictate the behavior and evolution of a system. For instance, Newton’s laws of motion govern the movement of a pendulum. Constraints, on the other hand, refer to the limits within which a system operates. These could be initial or boundary conditions, or operational constraints such as force or fuel limits. The last component of an optimization task is the goal. The goal sets the ultimate aim of the optimization, be it to reach a certain physical state or to minimize costs. Each kind of goal brings its own unique challenges, and hence demands a tailored approach for optimal solutions.

To resolve these optimization problems, traditional numerical methods have been predominantly used, yet they can be computationally expensive and may struggle with complex systems or constraints. Gradient descent or simplex methods are often used in real-world optimization tasks for minimizing duration time or cost, and in particular, the gradient descent method is actively used even in abstract tasks such as the optimization of internal parameters in machine learning. In large and complex real-world systems with expansive search space, genetic algorithms (GA) can be used, but they are computationally expensive as they require evaluating many potential solutions in each generation. Recently, optimization and control techniques utilizing deep reinforcement learning (RL) have been applied in various fields^1–4^. For example, it has been used in optimizing semiconductor chip design^5,6^ and actuation scenarios for fusion reactors^7–10^, which are some of the most complex engineering systems. However, using RL, even in mathematically well-defined systems, typically requires millions of iterations to find the optimal scenario. Additionally, tasks that require temporally extended planning strategies or unstable scenario solutions are still challenging to achieve the final goal with RL^1,11,12^.

In this study, we introduce a new approach to real-world optimization tasks using a physics-informed neural network (PINN). Specifically, we demonstrate that it can find the optimal scenario more efficiently in well-defined engineering systems than RL techniques. We showcase the optimization using PINN in different tasks: (1) Swinging up a pendulum to reach the goal state, (2) determining the shortest-time path connecting two given points, and (3) finding a minimal-thrust swingby trajectory for a spacecraft.

### Physics-informed neural computing for real-world optimization tasks

Physics-informed neural network (PINN) is a recent advancement in the field of deep learning that leverages the power of neural networks to solve differential equations and learn the underlying physics of a given problem^13,14^. The main idea behind PINN is to incorporate governing physics laws as another objective function along with given sparse data like boundary conditions during the training process of the neural network. By incorporating physics laws into the learning process, PINN can efficiently predict complex physical phenomena from sparse or absent data. It is rapidly emerging as an alternative scheme to traditional computational simulation techniques^15–19^. The rise of PINN is attributed to its ability to operate without the use of a mesh and to easily implement arbitrary constraints beyond initial or boundary conditions.

**Figure 1:**
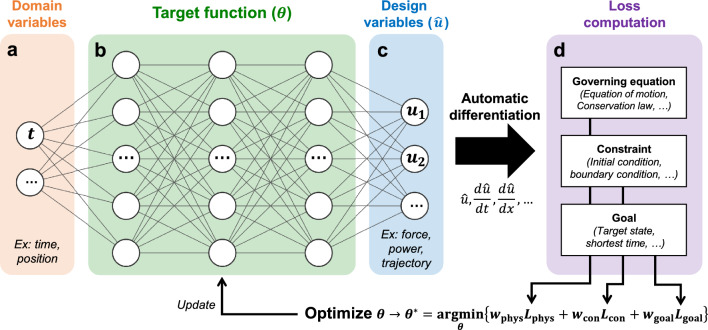
Neural network architecture using physics-informed loss to solve the optimization task. (**a**) The domain variables (ex. time or position) as neural network inputs. (**b**) The target function to be optimized ($\theta$), composed of multi-layer perceptrons. (**c**) The design variables as neural network outputs. (**d**) The loss functions (physics loss, constraint loss, and goal loss) which are weighted-summed for the final objective function.

Though initially used for solving physics equations unrelated to optimization tasks, PINN possesses potential features to be applied to a broader range of tasks. One of the potentials of PINN is its ability to integrate an additional objective function beyond governing physics laws and constraints. In the case of optimization tasks like our study, by integrating a penalty for violations of the desired goal into the objectives, PINN can find the optimal scenario that not only satisfies the given governing laws and constraints but also reaches the goal. Figure 1 illustrates the architecture of PINN, which finds a solution by setting the governing laws, constraints, and goals of the given task as objective functions.

The main target function we wish to find (the neural network $\theta$, Fig. 1b) is a function that maps the optimal path of design variables (Fig. 1c) within a given domain (Fig. 1a). Domain variables could be time, position, or available budget, depending on the given task. Design variables might be scenarios of force or power over time, or spatial coordinates of a trajectory. By constructing the target function with a neural network, where all the nodes are differentiable, the exact derivatives of the design variables with respect to the domain can be easily obtained with automatic differentiation. Using the computed design variables and their derivatives through the neural network $\theta$, we can estimate how well the governing equations, constraints, and goals are satisfied. Figure 1d illustrates the calculation of the losses through Eqs. (1) (The physics loss, $L_{phys}$) and (2) (The constraint loss, $L_{con}$) from the neural network outputs. The goal loss, $L_{goal}$, shown in Fig. 1d is determined according to the given task.

1 $$\begin{aligned}{} & {} L_{phys}(\theta )=\frac{1}{N_{\Omega }}\sum _{j=1}^{N_{\Omega }} \Vert {\mathcal {F}}\left( t_j; \theta \right) \Vert _2^2 \text { for } t_j \in \Omega . \end{aligned}$$

2 $$\begin{aligned}{} & {} L_{con}(\theta )=\left\{ \begin{array}{cc} \frac{1}{N_{\partial \Omega }}{\sum }_{j=1}^{N_{\partial \Omega }} \Vert {\hat{u}}(t_j; \theta )-BC \Vert _2^2 \text { (Dirichlet) } \\ \frac{1}{N_{\partial \Omega }}{\sum }_{j=1}^{N_{\partial \Omega }} \Vert \frac{d{\hat{u}}}{dt}(t_j; \theta )-BC \Vert _2^2 \text { (Neumann) } \\ \end{array} \right. \text { for } t_j \in \partial \Omega . \end{aligned}$$

Here, $\Omega$ is the domain of interest and $\partial \Omega$ is its boundary. $N_{\Omega \text { or } \partial \Omega }$ is the sampling counts on each domain. ${\mathcal {F}}$ is the given governing equations and *BC* is the given boundary conditions, which could be Dirichlet, Neumann, or any custom boundary conditions. Through the estimated loss values, the function of solution path ($\theta ^*$) can be determined with the converging process in Eq. (3). Here, $w_{phys \text {, } con \text {, or } goal}$ is the weight for each loss value.

3 $$\begin{aligned} \theta \rightarrow \theta ^* = {\text {argmin}}_\theta \{w_{phys}L_{phys}(\theta )+w_{con}L_{con}(\theta )+w_{goal}L_{goal}(\theta )\}. \end{aligned}$$

The main distinction from traditional PINN solving differential equations is the objective function concerning the goal state, $L_{goal}$. By appropriately defining $L_{goal}$, it can be applied to various real-world optimization problems, such as achieving a given target state, minimizing consumed cost or time, or finding an optimal path. Specifically, PINN works effectively for well-defined problems through governing equations, although there are approaches even if the governing laws are unknown, which will be discussed later. The forms of the loss functions may vary depending on the characteristics of the problem, and in some cases, the constraint loss or the goal loss may be integrated into the governing equations. In the following sections, we will showcase how these are composed in different optimization tasks.

### Swinging up a pendulum

**Figure 2:**
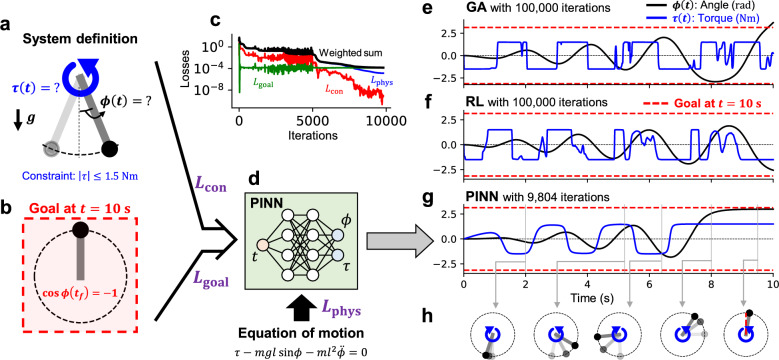
Application of PINN for optimizing the torque scenario in swinging up a pendulum. (**a**) The description of the fixed-axis pendulum, where the design variables are the angle $\phi$ and the torque $\tau$ as functions of time *t*. (**b**) The goal state in this optimization task, $\cos {\phi }=-1$ at $t=10 \text { s}$. (**c**) The history of loss values over iterations. (**d**) The illustration of the neural network incorporating the equation of motion into its objective function. The input is *t* and the outputs are $\phi$ and $\tau$. (**e**) A baseline result of a GA algorithm. (**f**) A baseline result of an RL algorithm using TD3. Both GA and RL produce wiggling torque scenarios. (**g**) The result of PINN, which determines swinging the pendulum back and forth to accumulate its energy to reach the goal. (**h**) Several snapshots of swinging up the pendulum.

Swinging up a fixed-axis pendulum is a well-known problem for testing the performance of a controller. Classical algorithms such as PID controllers^20^ also work well for swing-up control of a pendulum. However, if the maximum available torque is limited, and it is grossly insufficient to invert the pendulum at once, PID or greedy methods hardly succeed in inverting it. In this section, we have taken the task of a fixed-axis pendulum shown in Fig. 2a where the torque magnitude ($\tau$) is limited by $|\tau | \le 1.5 \text { Nm}$. In this task, the goal is to reach a vertically inverted state at $t=10 \text { s}$, as shown in Fig. 2b; in other words, $\cos {\phi (t=10 \text { s})}=-1$ with the angle from the rest state, $\phi$. Here, we empirically know that reaching this goal requires a temporally extended and nonlinear scenario accumulating energy through multiple swings in different directions.

In this system, the task is to find the torque scenario to reach the goal state shown in Fig. 2b, with a low-torque actuator. The governing equation (${\mathcal {F}}$ in Eq. (1)) is the equation of motion under gravity,

4 $$\begin{aligned} {\mathcal {F}}=ml^2 \ddot{\phi } - (\tau - mgl\sin {\phi }). \end{aligned}$$

Here, $m=1 \text { kg}$ is the mass of the ball of the pendulum, $l=1 \text { m}$ is the length of the massless string, and $g=9.8 \text { m/s}^2$ is the gravitational acceleration. The constraints (Eq. 2) are the initial conditions with angle ($\phi$) and angular velocity (${\dot{\phi }}$) being zero. If we use a greedy method like a proportional controller, it determines a unidirectional constant maximum torque command, trying to reduce the error from the goal. However, since the available torque magnitude is grossly small, a unidirectional swing alone cannot invert the pendulum. A solution must be found that can reach the goal in the long term, even if it momentarily moves away from it. GA or RL that use the sum of long-term discounted rewards can exhibit better performance in such problems that require long-term planning. Figure 2e and f show the scenarios of $\tau$ and the resulting trajectories of $\phi$, obtained by using GA and RL, respectively. Here, the state variables are set as the angle $\phi$ and the angular velocity ${\dot{\phi }}$, and the action variable is set as the torque ($-1.5\le \tau \le 1.5$). For the RL, the TD3 algorithm^21^ was implemented, and the reward was set as $R=-\left( \cos {\phi }-(-1)\right) ^2$. More settings for the GA and RL baselines can be found in the "Methods" section. After $10^5$ iterations of training, both GA and RL are observed to approach the inverted state of the pendulum ($\cos {\phi }=-1$) at $t=10 \text { s}$. However, since both GA and RL start at $t=0 \text { s}$ and proceed to the final state through random mutation and exploration (“bottom-up”) during training process, they produce a wiggling torque scenario due to meaningless exploration during intermediate periods that don’t significantly influence the final goal. Here, at the inference phase, the random exploration has been turned off, and the wiggling scenario is not due to the action noise. Introducing additional rewards or regularization to mitigate this wiggling leads to additional issues such as defining a metric to quantify the wiggling, deciding weights for different objectives, and dealing with trade-offs arising from multi-objectives.

On the other hand, to solve this torque scenario optimization task using PINN, the goal loss is defined as $L_{goal}=\left( \cos {\phi (t=t_f)}-(-1)\right) ^2$, with the target time $t_f=10 \text { s}$. The neural network input (Fig. 1a) is set to time *t*, and the output (Fig. 1c) is set to angle $\phi$ and torque $\tau$, as shown in Fig. 2d. While GA or RL decide on an action based on the state at every time step, sequentially generating a scenario from $t=0$ to 10 s, PINN takes the time variable as an input and outputs the scenario over time in a single pass. Through such an I/O approach, PINN potentially allows for more efficient inference in tasks designing a trajectory over time, rather than real-time decision-making. To impose a limitation on the torque size, a tanh activation is applied only to the torque output. A detailed description of the system, hyperparameters, and numerical environments can be seen in the "Methods" section. The evolutions in loss values during iterative updates through Eq. (3) are depicted in Fig. 2c. After 9804 iterations, the training becomes converged with $L_{phys}<10^{-5}$, $L_{con}<10^{-9}$, and $L_{goal}<10^{-4}$. Considering each term in Eq. (4) is *O*(1), $L_{phys}<10^{-5}$ indicates a high enough level of physical fidelity. The solution at the converged iteration can be seen in Fig. 2g. With limited torque, PINN has derived a scenario that effectively accumulates energy by swinging the pendulum back and forth and attains a force balance at the goal state. Compared to the baselines in Fig. 2e and f, it can be observed that PINN produces a smoother scenario to reach the goal with fewer modulations of torque direction. It is worth noting that neither PINN nor the baselines were given a goal of minimal modulations. Snapshots of the pendulum during this process can be seen in Fig. 2h.

We also observed that the training of PINN proceeds in a non-chronological and top-down manner. GA and RL operate in a chronological and bottom-up manner, where each episode progresses from $t=0$ to $t=t_f$, with the final state getting closer to the goal as episodes accumulate. In this case, learning a reward model and approaching the goal rely on trial-and-error exploration to understand the environment, thus there needs to be sufficient random noise or mutation to find better solutions. In PINN, however, the environment is directly informed by the mathematical form of governing equations. PINN then seeks an optimal solution based on a mathematical objective function that incorporates goal information as well, eliminating the need for many empirical trials and errors to explore the environment. As seen in Fig. 2c, $L_{goal}$ first converges to a sufficiently low level ($<10^{-4}$) from the early phase of iterations, followed by the decrease of $L_{con}$ and $L_{phys}$, exemplifying a top-down approach. Through this approach of PINN, even if the solution path is complex, it can be derived with fewer iterations than GA and RL, as shown in Fig. 2e–g. A more quantitative comparison of computational efficiency can be found in the "Methods" section (Table 2).

### Determining the shortest-time path connecting two given points

Many real-time optimization problems involve minimizing the cost or time taken to reach a goal. In this section, we test the PINN method on the task of finding the shortest-time path in several environments where an analytic solution exists. One significant difference from the previous example (swinging up a pendulum) is that the time taken is a variable and the subject to be minimized.

We present two famous examples of finding the shortest-time path. (1) Fermat’s principle, or the principle of least time, is that the path of the light ray between two given points is determined as the path that can be traveled in the shortest time. (2) Another one is the shortest-time descent path between two given points under gravity, known as the brachistochrone curve. It has been mathematically proven that it is part of a cycloid.

**Figure 3:**
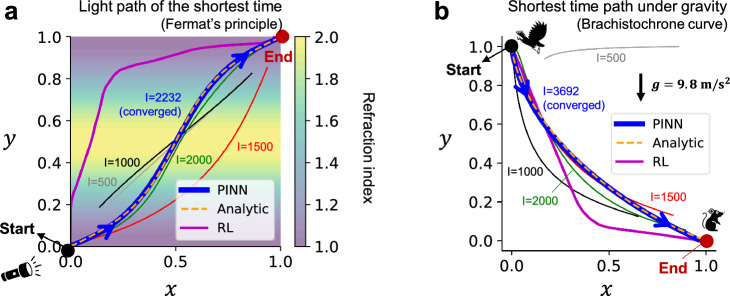
Applications of PINN for determining the shortest-time path under specific environments. (**a**) Finding the shortest-time path of a light ray within the medium where the refraction index varies along *y*. (**b**) Finding the shortest-time descent path between two given points under constant gravity. In the two figures, the analytic solutions are given in yellow dashed lines and the converged solutions by PINN are shown in blue. For baselines, the results using RL with $10^5$ iterations are shown in magenta lines.

The refraction of light can be explained by Fermat’s principle. When the refraction index in a medium varies according to the location, the speed of the ray changes. In this case, the path of the ray bends to find a shorter-time path in the medium. An example of the refraction of light within a medium where the refraction index varies is shown in Fig. 3a.

In the 2D *xy* space of Fig. 3a, the refraction index (*n*) has a sinusoidal profile with respect to *y*, as represented by the contour color. The ray slows down around $y=0.5$ where the *n* is high. Therefore, the path of light that passes in the shortest time will be determined to become more vertical near the slowest region, $y=0.5$. Thus, the ray trajectory will bend according to the changes in the refraction index, consistent with the analytic solution dictated by the law of refraction, as shown in yellow dashed line in Fig. 3a. When using RL to search for the shortest-time arrival trajectory, even after $10^5$ iterations of training, a trajectory far from the analytic solution emerges, as indicated by the magenta line. While this trajectory does become vertical around $y=0.5$ where *n* is high, it is still far from being the shortest possible time.

To find the shortest-time path using PINN, the input of the neural network of Fig. 1 is set to the normalized time ($t_N$), and the outputs are the *x* and *y* coordinates. The governing equation is given by ${\mathcal {F}}=\left( \frac{1}{T} \frac{dx}{dt_N} \right) ^2+\left( \frac{1}{T} \frac{dy}{dt_N} \right) ^2-\left( \frac{c}{n} \right) ^2$, where $c=1$ is the speed of light in vacuum and *T* is the time taken from the starting point to the endpoint. Here, *T* is unknown and a trainable variable during the neural network training. Constraints are set as the coordinates of the starting and ending points, respectively (0, 0), (1, 1), and the goal is set as $L_{goal}=T$, aiming to minimize the time taken. More specific settings can be found in the "Methods" section.

The solid lines in Fig. 3a depict the solution paths at each checkpoint (iteration $I=500, 1000, 1500, 2000$), with the blue solid line representing the converged final solution ($I=2232$). Unlike traditional methods that explore the shortest-time curve from a fixed start point to an endpoint, the PINN approach shows the characteristic of searching for the shortest-time curve while simultaneously approaching the start and endpoint. The converged solution shows a path that almost coincides with the analytic solution.

Figure 3b illustrates the search for the shortest-time descent curve (brachistochrone curve) connecting two given points under gravity ($g=9.8 \text { m/s}^2$). Here, the yellow dashed line is the analytic solution for the shortest-time curve, which is a cycloid. In the RL solution with $10^5$ iterations shown in magenta, the path more vertically free-falls to increase velocity and then changes direction horizontally at the lower altitude to move at a high speed. While this seems intuitively plausible, it deviates from the actual shortest curve, the cycloid. On the other hand, for PINN, the governing equation is set as the mechanical energy conservation law, ${\mathcal {F}}=gy_0-\left( gy+\frac{1}{2}\left( \left( \frac{1}{T} \frac{dx}{dt_N} \right) ^2+\left( \frac{1}{T} \frac{dy}{dt_N} \right) ^2 \right) \right)$, and the constraints were set using the start ($(x_0, y_0)=(0, 1)$) and endpoint coordinates ($(x_1, y_1)=(1, 0)$). The goal, similar to Fig. 3a, was set as $L_{goal}=T$ to find the shortest-time curve. In Fig. 3b, at the initial phase ($I=500$), a non-physical path that fails to align the start and endpoints is derived. But it gradually converges to a path close to the analytic solution shown in yellow. This approach using PINN to search for the shortest-time path can be utilized in chip design to find the minimum-loss circuit.

### Finding a swingby trajectory of a spacecraft

**Figure 4:**
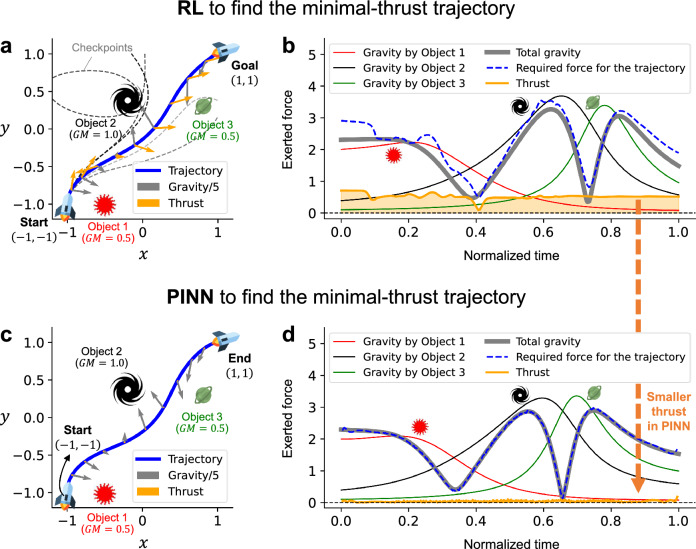
Application of RL and PINN for finding the swingby trajectory of a spacecraft with minimal additional thrust. (**a**) The description of the system with three astronomical objects and the converged solution path of the spacecraft swingby obtained by RL after $10^5$ iterations. 1/5 of the gravity and the thrust are shown in the arrows of gray and yellow, respectively. (**b**) The magnitudes of the gravitational forces exerted by each object, total gravity, thrust, and the required force to follow the trajectory determined by RL. (**c**) The converged solution path of the spacecraft swingby obtained by PINN after 3215 iterations. (**d**) The magnitudes of the gravitational forces exerted by each object, total gravity, thrust, and the required force to follow the trajectory determined by PINN.

The last example is a case where the goal can be integrated into the governing equation. We present the problem of finding the swingby trajectory of a spacecraft that can reach the given destination using the least amount of thrust, leveraging gravity. In a situation with multiple astronomical objects, the spacecraft experiences dynamic gravity depending on its position. The goal is to find a path that allows the spacecraft to reach its target position without using its onboard fuel, utilizing these gravitational forces. In this problem, even a slight deviation from the solution path can sensitively change the gravity acting on the spacecraft, resulting in a completely different path and destination, like falling into a star. This is an example of a problem with a very narrow and unstable solution, and it has been challenging to find the narrow solution path using random exploration or mutation with RL or GA. Figure 4a shows three different astronomical objects located at positions $(x_o, y_o)=(-0.5, -1.0), (-0.2, 0.4), (0.8, 0.3)$ and having masses multiplied by the gravitational constant $GM_o=0.5, 1.0, 0.5$.

To address this problem, we first consider the use of the RL approach. Here, the RL agent receives the current position coordinates (*x*, *y*) and decides on the *x* and *y* components of the thrust. Each episode starts from the position $(x_0, y_0)=(-1, -1)$ and terminates when the spacecraft reaches $x=x_0 \pm 2$ or $y=y_0 \pm 2$, at which point the final reward is given by $R=-Mean(|\text {Thrust}|^2) - ((x-1)^2 + (y-1)^2)$. More detailed RL settings can be found in the "Methods" section. The blue solid line in Fig. 4a represents the path of the spacecraft obtained using RL over $10^5$ iterations. The gray arrows on the trajectory signify 1/5 of the gravity at each point, while the yellow arrows indicate the additional thrust required to follow that path. The results of the checkpoint models every 20,000 iterations during the learning process are represented by the black dashed lines, with darker colors indicating later checkpoints. As seen from the checkpoint results, even though the spacecraft starts at the same position with the same velocity, the final trajectory can vary sensitively depending on the thrust scenario. The blue path, the final result, derived a thrust scenario that reaches the final destination of (1, 1), but it’s evident that its magnitude is still substantial. Figure 4b shows the magnitudes of the gravitational forces exerted by each object with respect to the normalized time, in addition to the total gravity and thrust. The magnitude of the thrust needed to follow the trajectory derived by RL is relatively small compared to gravity, but it still possesses a magnitude $>0.5$. Here, more increasing the total learning iterations in RL couldn’t dramatically reduce the required thrusts.

On the other hand, to apply PINN for this task, the neural network input is the normalized time $t_N$, while the normalization factor *T* (or the time taken from the starting point to the endpoint) is a trainable variable, and the outputs are the *x* and *y* coordinates for the swingby trajectory. Constraints are set as the coordinates of the starting and ending points, respectively $(-1, -1)$, (1, 1). The goal in this task can be integrated with the governing law by setting the thrust (Eq. 5) as ${\mathcal {F}}$ for the physics loss.

5 $$\begin{aligned} {\mathcal {F}}=\text {Thrust vector}=\left\{ \begin{array}{cc} \frac{1}{T^2}\frac{d^2x}{dt_N^2}-{\sum }_{(x_o, y_o, GM_o)} \left( \frac{-GM_o (x-x_o)}{\left( (x-x_o)^2+(y-y_o)^2 \right) ^{1.5}} \right) \text { (x component of thrust) } \\ \frac{1}{T^2}\frac{d^2y}{dt_N^2}-{\sum }_{(x_o, y_o, GM_o)} \left( \frac{-GM_o (y-y_o)}{\left( (x-x_o)^2+(y-y_o)^2 \right) ^{1.5}} \right) \text { (y component of thrust) } \\ . \end{array} \right. \end{aligned}$$

Equation (5) represents the *x* and *y* components of the thrust vector required to follow a given path (*x*, *y*), derived from the equations of motion. By setting these expressions as a function ${\mathcal {F}}$ in Eq. (1), the objective function to minimize, PINN can find the spacecraft’s path where ${\mathcal {F}}$ approximates 0 at every point along the path. This solution not only satisfies the equations of motion under gravity but also minimizes the magnitude of thrust required to follow the path. The blue solid line in Fig. 4c shows the converged path of the spacecraft through this method, after 3,215 iterations. The gray arrows represent 1/5 of the gravity acting at each point, while the yellow arrows indicate the additional thrust required to follow that path. Figure 4d shows the magnitudes of the gravitational forces exerted by each object with respect to the normalized time $t_N$, in addition to the total gravity and thrust. The required thrust is negligible compared to gravity, and the acceleration needed to follow the path (shown by the blue dashed line in Fig. 4d) almost exactly matches the total gravity acting on the spacecraft. This means that, if the spacecraft moves along the obtained solution path, it can reach the target point (1, 1) solely guided by gravity, almost without using its own thrust.

## Discussion

In this study, we introduce a new approach using PINN to perform real-world optimization tasks. We have validated this method on well-known test problems with different characteristics: Swinging up a pendulum, Fermat’s principle, the brachistochrone curve, and the swingby of a spacecraft. These results demonstrate that PINN can be applied to optimize the trajectory or scenario in well-defined systems.

In most optimization tasks, such as the examples in our work, there is a need to find the trajectory of intermediate steps that satisfy a given desired final state - making it an inverse problem. Such inverse problems are often ill-posed, with solutions that are highly unstable and narrow or highly sensitive to changes in the final state^22^. Traditional optimization solutions often address this by converting it into multiple forward problems. For instance, other machine learning techniques such as RL and GA deal with each episode as a series of chronological action-response tasks that build up to reach the final state (forward problem), subsequently adjusting the policy through these tasks. This approach, however, brings its own set of drawbacks such as the exploration-exploitation trade-off^11^. In contrast, PINN effectively works with inverse and ill-posed problems^23–25^. In other words, instead of chronological interactions with the environment, PINN intrinsically integrates information about the environment (via the governing equation) and the goal, optimizing trajectories within the input domain non-chronologically.

Therefore, PINN doesn’t learn about the environment through explorative trial and error (as RL or GA do) but is informed directly via the governing equation in its mathematical form. Thus, there’s less need for exploration to understand the environment, yielding fewer iterative learning for convergence as shown in Fig. 2. Moreover, in tasks that aren’t about real-time decision-making but rather designing trajectories or scenarios, PINN is more efficient as it can deduce them in only a single pass. (In RL approaches, the trajectory is built up by deciding actions sequentially at every time step.) While RL is more suited than PINN for real-time control^8^, chatbot^26^, or competitive games^27^ where interactive decision-making is needed in various states, PINN can outperform for a single optimization task that requires finding a narrow solution path. In future research, a quantitative analysis with model-based RL will also be needed, which is more suitable in cases like this study where a clear model is provided.

However, PINN does face a challenge in that it can struggle in systems where governing laws are unknown and thus cannot define the physics loss in Eq. (1). In many real-world, industry-level optimization problems, the governing law may be unknown or difficult to express mathematically. For example, in nuclear fusion reactors, one of the most advanced engineering systems, many phenomena are not explainable by physical theories^28^. In our previous study on RL-based optimization of fusion reactor operation scenarios, to address this issue, we used a surrogate model composed of experimental data-driven neural networks as a training environment^7^. Similarly, in the case of PINN, a neural network surrogate model could be used to implement physics loss instead of governing laws expressed with differential equations. Especially since the neural network of this surrogate model is also composed of differentiable nodes, it can play a similar role to the differential equation through Eq. (3). By using PINN optimization with a surrogate model that describes fusion plasma physics, we will be able to search the optimal fusion reactor operation scenario for efficient nuclear fusion energy production, which is part of our future work.

## Methods

### Common numerical environment

To implement PINN for optimization tasks, we utilized the DeepXDE library^14^. For the optimization of the neural network in the PINN framework, the Adam^29^ and L-BFGS^30^ algorithms were applied sequentially. All instances of the neural network in our work are designed with the same structure, consisting of three hidden layers, each with 64 nodes. Hyperbolic tangent (tanh) activation is applied to each hidden layer to provide nonlinearity. Table 1 summarizes the governing laws, constraints, and goals used in the optimization examples presented in this study. The detailed settings for numerical environments and the Python scripts can be found at https://github.com/jaem-seo/pinn-optimization.

**Table 1:** Description of the optimization examples presented in this study.

	Swinging up a pendulum	Fermat’s principle	Brachistochrone curve	Swingby of a spacecraft
Governing law	Equation of motion	Constant light speed	Conservation of mechanical energy	Equation of motion
Constraints	Initial angle, angular speed, and torque	Initial and final coordinates	Initial and final coordinates	Initial and final coordinates
Goal	Inverted state	Shortest duration time	Shortest duration time	Zero thrust
Note	Fixed target time	Variable duration time	Variable duration time	Integrated goal and governing law
Epochs for Adam	5000	2000	2000	2000
Learning rate for Adam	0.02	0.001	0.001	0.001
Weights	(1, 10, 1)	(1, 1, 0.01)	(1, 1, 0.01)	(1, 1, −)
Output transform	tanh (only for $\tau$)	Sigmoid	Sigmoid	tanh

The hyperparameters shown are for PINN.

For the baseline results using RL, we used the TD3 algorithm^21^ with the Stable-Baseline3 library^31^. All the RL agents shown in this work are composed of three hidden layers, each with 64 nodes, and trained for $10^5$ iterations.

Table 2 shows the comparison of elapsed wall time using each machine learning method in the four examples shown in Figs. 2, 3 and 4. For these computations, an RTX-3080Ti GPU was utilized.

**Table 2:** The elapsed wall time taken for each machine learning method in the four examples shown in Figs. 2, 3 and 4.

	PINN	RL	GA
Swinging up a pendulum	44±16	892±10	4015±34
Fermat’s principle	45±29	887±7	–
Brachistochrone curve	36±11	923±8	–
Swingby of a spacecraft	62±17	906±12	–

The average and the standard deviation of five ensembles are shown for the wall time. The unit of the wall time is seconds.

### Setting for swinging up a pendulum

To solve the torque scenario that reaches the inverted state of a pendulum by using PINN, the outputs of the neural network are set as ${\hat{u}} = \{\phi , \tau _{logit} \}$, with the angle from the rest $\phi$ and the torque logit $\tau _{logit}$. To limit the maximum magnitude of the torque $\tau$, the torque logit is transformed via the tanh function, and then multiplied by the maximum available torque ($1.5 \text { Nm}$). The governing equation (${\mathcal {F}}$ in Eq. (1)) to describe the motion of a pendulum under gravity is shown in Eq. (4). As constraints, the initial conditions for *BC* in Eq. (2) are given, as in Eq. (6), representing a state of rest.

6 $$\begin{aligned} \{ \phi , {\dot{\phi }}, \tau \} = \{0, 0, 0 \} \text { at } t=0 \text { s}. \end{aligned}$$

To make PINN find the torque scenario that reaches the inverted state of the pendulum at $t=10 \text { s}$, the goal loss $L_{goal}$ is defined as in Eq. (7).

7 $$\begin{aligned} L_{goal}=\left( \cos {\phi (t=10 \text { s})}-(-1)\right) ^2. \end{aligned}$$

The physics, constraints, and goal losses obtained through Eqs. (4), (6) and (7) are weighted-summed into a final objective function through the weights $\{w_{phys}, w_{con}, w_{goal}\}=\{1, 10, 1\}$. For the solution of PINN using this, Adam and L-BFGS optimizers were used sequentially. Adam was applied for 5000 epochs at a learning rate of 0.02, after which L-BFGS was applied for 4,804 iterations until convergence, as shown in Fig. 2c.

For the RL baseline (shown in Fig. 2f) to compare the performance of PINN, an RL model with observation variables $S=\{\phi , {\dot{\phi }} \}$ and an action variable $A=\{\tau \}$ was used. The reward is set as the negative value of $L_{goal}$ in Eq. (7), which is given each steps densely. The RL agent was trained for 100,000 iterations through TD3^21^, implemented by Stable-Baselines3^31^. The GA baseline shown in Fig. 2e has been obtained by PyGAD^32^ with the same environment as RL. For GA, the number of total learning steps has been determined to be the same as the others: 100,000 learning steps = 25 generations $\times$ 4 parents $\times$ 1000 steps per generation.

**Figure 5:**
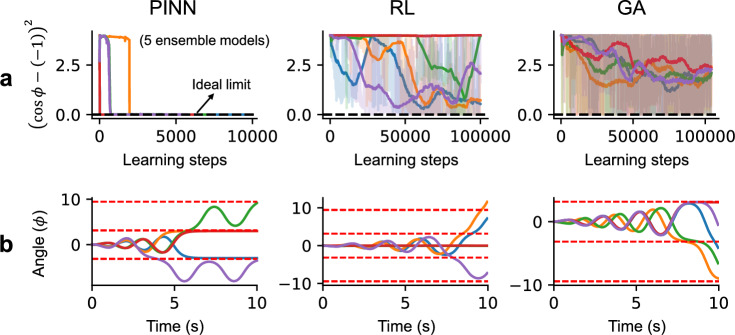
The variation among five ensemble models in PINN, RL, and GA. (**a**) The learning curve variation. For the RL and GA curves, smoothed lines are shown in solid lines, while the original curves are indicated with transparent colors. (**b**) The variation of inference results after the training, without exploration noise. The goal states ($cos\phi =-1$) are also shown in red dashed lines.

Figure 5 shows the learning curves when using PINN, RL, and GA. The variations among the ensemble models with 5 different random seeds are also shown in each plot. Since different machine learning methods use different metrics as the cost function, here we compare the difference between the achieved state and the goal, shown in Eq. (7). In Fig. 5a, the variation in ensemble models for PINN is smaller than the other methods. Although all three methods successfully find the scenarios for accumulating the pendulum energy, RL or GA hardly match the goal state at the final point, as shown in Fig. 5b.

### Setting for determining the shortest-time path connecting two given points

To determine the shortest-time path by using PINN, the outputs of the neural network are set to ${\hat{u}} = \{x, y \}$, the coordinates with respect to the normalized time $t_N$. In these tasks, we constrain the range of the path within $0\le x\le 1$ and $0\le y\le 1$, thus, the last activation function is set to the sigmoid function. The governing equations for Fig. 3a and b are shown in Eqs. (8) and (9), respectively.

8 $$\begin{aligned}{} & {} {\mathcal {F}}=\left( \frac{1}{T} \frac{dx}{dt_N} \right) ^2+\left( \frac{1}{T} \frac{dy}{dt_N} \right) ^2-\left( \frac{c}{n} \right) ^2 \end{aligned}$$

9 $$\begin{aligned}{} & {} {\mathcal {F}}=gy_0-\left( gy+\frac{1}{2}\left( \left( \frac{1}{T} \frac{dx}{dt_N} \right) ^2+\left( \frac{1}{T} \frac{dy}{dt_N} \right) ^2 \right) \right) . \end{aligned}$$

The constraints are given by the boundary conditions Eq. (10), where $(x_0, y_0)$ and $(x_1, y_1)$ are shown with start and end points in Fig. 3.

10 $$\begin{aligned} (x, y)=\bigg \{ \begin{array}{cc} (x_0, y_0) \text { at } t_N=0 \\ (x_1, y_1) \text { at } t_N=1 \\ \end{array} \end{aligned}$$

For the goal of minimizing the time taken, the goal loss is defined as $L_{goal}=T$. The weights for the losses are set to $\{w_{phys}, w_{con}, w_{goal}\}=\{1, 1, 0.01\}$, which prioritize satisfying the governing equation and the boundary conditions over reducing the time taken. Adam was applied for 2000 epochs at a learning rate of 0.001. Then, L-BFGS was applied for 1232 and 2692 iterations until convergence in Fig. 3a and b, respectively.

For the RL baselines, the observation variables $S=\{x, y\}$ and an action variable $A=\{ \arctan {(dy/dx)}\}$ are set. The reward of $R=-w_T T - ((x-x_1)^2 + (y-y_1)^2)$ is given at the termination when the position reaches $x=x_0 \pm 1$ or $y=y_0 \pm 1$, where the weight for the punishment on the time taken $w_T$ is set $10^{-2}$ and $10^{}$ for Fig. 3a and b, respectively.

### Setting for finding a swingby trajectory of a spacecraft

To finding the minimal-thrust swingby trajectory of a spacecraft by using PINN, the neural network outputs are set to ${\hat{u}} = \{x, y \}$, similarly to the previous example. Here, the starting point is $(x_0, y_0)=(-1, -1)$ and the endpoint is $(x_1, y_1)=(1, 1)$, and the last activation function is set to the tanh function. The governing equations under universal gravitation with three astronomical objects are shown in Eq. (5), and the constraints are given by the boundary condition, Eq. (10). The weights for the losses are set to $\{w_{phys}, w_{con}\}=\{1, 1\}$, where the goal loss is included in the physics loss. Adam was applied for 2000 epochs at a learning rate of 0.001. Then, L-BFGS was applied for 1215 iterations until convergence.

For the RL baseline, the observation variables $S=\{x, y, {\dot{x}}, {\dot{y}}\}$ and action variables $A=\{ \text {Thrust}_x, \text {Thrust}_y \}$ are set. The reward of $R=-Mean(|\text {Thrust}|^2) - ((x-x_1)^2 + (y-y_1)^2)$ is given at the termination when the position reaches $x=x_0 \pm 2$ or $y=y_0 \pm 2$.

## Data Availability

The Python scripts producing the data that support the findings of this study are openly available at https://github.com/jaem-seo/pinn-optimization.
